# Retaining Artificial Dentures in the Mouth

**Published:** 1896-10

**Authors:** Joseph Spyer


ARTICLE VIII.

### RETAINING ARTIFICIAL DENTURES IN THE MOUTH.

BY JOSEPH SPYER, M. D., D. D. S.,

It is now nearly forty years since I began to practice dentistry, having commenced in 1857, and during that period I have introduced various methods for the retention of artificial dentures in the mouth. The air chamber which has been so long in vogue has never seemed to me to meet the requirements.

As far back as 1861 I used in my practice, with a moderate degree of success, a gold plate made in two parts; the first plate, which held the teeth, being of solid metal and very narrow, to which was attached on its inner side a second plate, perforated with numerous small holes, thus causing the denture to hold in the mouth by capillary attraction.

In 1862 I discovered a method for retaining rubber plates, which was also used with varying success. It comprised making a square groove on the plaster model, about an inch back of the alveolar ridge, up to which square groove, which was possibly an eighth of an inch in width, the model was trimmed away. After the plate was vulcanized, there was on its inner edge, on the palatal side, a narrow projection corresponding to the square groove made in the model, a sort of narrow ridge elevated above the surface of the plate, which produced a suction.

In 1885 I introduced the surface cohesive forms, which consist of a thin sheet of block tin, having small rounded depressions. This is placed on the plaster model, and, after vulcanization, the entire plate is found covered with small rounded projections on the palatal side, which hold it firmly in the mouth.

But I was not entirely satisfied, and in 1894 I invented the automatic suction cavity, which consists of sheet block tin stamped out to represent a form of suction running the entire length of the plaster model. It makes possible the use of a very narrow plate. The block tin form, which makes the cavity, has two narrow, parallel slots throughout its entire length, which produce on the palatal surface of the plate two corresponding parallel ridges, in addition to the suction form.

My latest method for retaining artificial dentures in the mouth, the Adhesive Plate, I believe, approaches the ideal. It causes the denture to adhere firmly in any mouth, hard or soft, high or flat, imparts a soothing sensation to the gums and mucous membrane, and prevents the irritation which arises from the hard rubber pressing upon soft tissues.

Many attempts have been made to provide a soft or elastic base for hard-rubber plates. in order to render them adhesive; but it has been found that the soft-rubber lining, or base, soon rots, thus leaving the denture practically worthless.

My invention overcomes the objection. I have discovered that with certain gums, found in South America, an adhesive plate can be made which will not deteriorate. It adheres firmly in any mouth, and at the same time provides a soft and elastic connecting medium, comforting to the gums and mucous membrane.

Dr. C. C. Richardson, of New York city, and many others among my colleagues, have succeeded by their employment in making dentures hold firmly in mouths where an ordinary hard-rubber plate would not serve at all.

The method of its use is very simple : After the case is flasked or packed, the Adhesive Plate is placed on the palatal surface of the plaster model, and the case vulcanized in the usual way. After vulcanization the Adhesive Plate will be found to be incorporated with the rubber on the palatal side.

My many years of practical experience in the insertion of artificial dentures in difficult mouths has demonstrated that my Adhesive Plate is superior to every other method of retaining artificial dentures in the mouth.—"Items of Interest."

